# On the Use of Chloroform and Ether in Puerperal Insanity

**Published:** 1849-07

**Authors:** 


*On the use of Chloroform and Ether in Puerperal Insanity.*—"As it is a great object to break the *continuance* of the sleeplessness of insanity, the occasional use of the *chloroform* vapour will be found valuable. We have had an opportunity of seeing more than one case in which it not only induced sleep, which had previously been absent for four or five nights and days, but the patient on recovering from its effects, was found to be quite tractable and free from violence. The inhalation of ether had been tried by M. Cazenave of Pau, in the case of a lunatic female who had rested neither night nor day for five months, and in which it induced tranquillity. M. Jobert, in a similar case, exhibited it with the good effect of inducing sleep, and restoring, temporarily, a state of rationality. M. Bouvier tried ether, also, in a case of puerperal mania, with very beneficial results. In this case there had been no sleep for a fortnight before using the ether; its use was followed on two occasions by '*un calme de quelques heures.*' We are bound, however, to add, that in some cases in which it had been tried by other practitioners, no beneficial effect was produced."—*Journal of Psychological Medicine.*

